# Tracking Foot Drop Recovery Following Lumbar-Spine Surgery, Applying Multiclass Gait Classification Using Machine Learning Techniques

**DOI:** 10.3390/s19112542

**Published:** 2019-06-04

**Authors:** Shiva Sharif Bidabadi, Tele Tan, Iain Murray, Gabriel Lee

**Affiliations:** 1 School of Civil and Mechanical Engineering, Curtin University of Technology, Perth 6102, Australia; T.Tan@curtin.edu.au; 2 School of Electrical Engineering, Computing and Mathematical Sciences, Curtin University of Technology, Perth 6102, Australia; I.Murray@curtin.edu.au; 3 St John of God Subiaco Hospital Perth 6008, Australia and School of Surgery of University of Western Australia, Perth 6009, Australia; glee.neurosurgery@bigpond.com

**Keywords:** foot drop, gait classification, machine learning, inertial measurement unit

## Abstract

The ability to accurately perform human gait evaluation is critical for orthopedic foot and ankle surgeons in tracking the recovery process of their patients. The assessment of gait in an objective and accurate manner can lead to improvement in diagnoses, treatments, and recovery. Currently, visual inspection is the most common clinical method for evaluating the gait, but this method can be subjective and inaccurate. The aim of this study is to evaluate the foot drop condition in an accurate and clinically applicable manner. The gait data were collected from 56 patients suffering from foot drop with L5 origin gathered via a system based on inertial measurement unit sensors at different stages of surgical treatment. Various machine learning (ML) algorithms were applied to categorize the data into specific groups associated with the recovery stages. The results revealed that the random forest algorithm performed best out of the selected ML algorithms, with an overall 84.89% classification accuracy and 0.3785 mean absolute error for regression.

## 1. Introduction

Walking ability is a key physical behavior that can strongly influence the individual’s personal independence, and therefore, the successful execution of their daily activities. Thus, assessment of the gait is frequently required in the clinical setting. It becomes increasingly necessary and important to assess the gait during the treatment of a wide range of gait disorders [1].

Regardless of the significance of gait disorders, there is no widely accepted method for assessing the quality of walking. The most common methods of gait evaluation are the Berg Balance Scale (BBS) [2], dynamic gait index [3], 10-Meter Walk Test [4], 6-Min Walk Test [5], and the Functional Ambulation Categories (FACs) [6]. All these methods evaluate walking ability using different ranges and through the application of different tasks and specified ranges [7]. During these tests, the medical practitioner visually observes the walking ability of the patient and ranks this ability. Therefore, the outcome of these assessments is subjective and may be inaccurate [8].

To overcome this limitation, different methods and devices have been investigated and introduced in practice. As a general measure, walking velocity has been used as an indication of gait health [9,10]. Manual muscle testers are also used to measure muscle strength, which is indirectly related to walking gait [11]. Recently, several studies have been conducted in an attempt to utilize the technology of various sensors in gait analysis. For example, the GAITRite System is a waking platform that uses a set of pressure sensors and a software system to track gait events [12]. The prosthetic activity monitor (PAM) is also used to assess physical activities based on acceleration measurements [1]. In addition, there are various complex optical motion capture systems, such as the Vicon system, that accurately measure human movements [13]. However, optical motion capture systems are expensive and require software expertise to operate; therefore, they are not practical for conducting daily clinical assessments [14]. Among the many different movement measurement methods, inertial measurement unit (IMU) sensors have been widely implemented for gait analysis due to their particular advantages, such as long-term monitoring and portable recording of ambulatory measurements [15,16,17]. 

The gait data collected from IMU sensors are generally large, noisy, complex, and interconnected. Automated methods (e.g., machine learning (ML), which can extract high-level information from raw data) are the preferred solution for managing this data characteristic [18,19]. These methods are currently being used in various medical applications [20]. For example, the walking-gait pattern of patients with Parkinson’s disease has been assessed for identification of gait freeze and for distinguishing the characteristics of Parkinson’s gait [21,22]. In addition, by utilizing ML algorithms, real-time monitoring of elderly patients’ fall down has become possible [23].

In other research, IMU sensors used together with ML analysis have been reported to assist in the identification of different gait phases and human identification via gait patterns [24,25]. These methods have also been implemented to detect gait-related symptoms, such as fall detection or lower extremity muscular fatigue [26,27]. The integration of ML algorithms for the analysis of IMU gait data has been found to be a feasible solution for helping in the diagnosis of neurological disorders involving gait [28].

Foot drop is a common gait disorder in adults, which may be due to varied aetiologies [29]. In simplistic terms, a weakness of the muscles which dorsiflex the foot at the ankle leads to a “dropping” effect on the front of the foot when an induvial walks. This can cause tripping and recurrent falls, with potentially disastrous consequences [30,31]. In the longer term, the abnormal gait pattern leads to compensatory mechanisms, which can also have an impact on other joints. It is important to recognise that the term, foot drop, is an all-encompassing end point phenomenon, which does not relate to a precise aetiology or pathophysiological mechanism. In this particular study, the authors have attempted to recruit patients who have developed a foot drop specifically due to compressive L5 (the fifth lumbar spine vertebrae) radiculopathy. An L5 lumbar nerve root lesion results in paresis of the tibialis anterior, extensor hallucis longus, extensor digitorum brevis, and gluteal muscles of the lower limb on clinical examination, and ankle dorsiflexion, ankle eversion, toe extension, and hip abduction weakness is typically documented [32]. Consequently, a characteristic foot drop and a pathological gait develops. This uniform criteria overcomes an obvious criticism of current studies, which purport to include patients with foot drop, but which, in reality, are attributable to heterogeneous pathologies. The present research aims to provide an objective methodology for tracking the recovery process in foot drop disorder, specifically in patients with L5 radiculopathy following lumbar-spine surgery. Also introducing a gait quality index based on regression technique to assist medical practitioners in the assessment of foot drop severity and the recovery state of patients. For this purpose, the gait characteristics were captured using IMU sensors and multiple ML algorithms were applied and results compared.

## 2. Materials and Methods

A series of walking trials were recorded using a custom designed system based on three IMU sensors [33]. The data were captured while walking from participants’ foot (barefoot), shank, and thigh in the form of different angle measurements over time (pitch (x), roll (y), and yaw (z)). This information was then transmitted and stored via wireless communication. This IMU system has been proven to be feasible for gait assessments in a previous study by the authors. That study compared the accuracy of the IMU system with the Vicon motion capture system (with an 18 camera setup) [31]. A strong correlation was observed (more than 96.9%) between the IMU system and the Vicon motion capture system [34].

### 2.1. Test Protocol

IMU sensors were attached to participants’ lower limbs using straps and double-sided tape. Participants were asked to perform three to seven walking trials in a straight line in their usual walking style. They were also asked to pause and wait for two seconds before walking. The first 500 ms of data were used to offset the sensor readings during the post-processing stage.

### 2.2. Participants

The gait data were gathered from two groups of participants having specified inclusion and exclusion criteria: (1) A group of 30 participants with healthy gait styles and without any reported gait-related problems (normal group); (2) a group of 56 patients recruited from a neurosurgery practice, who presented with ankle-dorsiflexion weakness with L5 radiculopathy origins (foot drop group). Degenerative lumbar spine disorders, such as radiculopathy at L5, can cause foot drop. The mechanism is through a compression of the nerve fibers that constitute the peroneal nerve [32]. The compressive pathology of the L5 nerve root was confirmed using magnetic resonance imaging (MRI) of the lumbar spine region.

The data from the second group were captured from the affected side of the lower limb and in three different stages: First, before the lumbar spine surgery (pre); second, during the first two days following surgery (post 1); and third, two to three weeks after the surgery while recovering (post 2).

The walking capabilities of different subjects at different stages may have varied due to wound pain, patient fatigue, or other related problems. Therefore, the speed, distance, and the number of steps in different trials were not equal. To overcome this limitation, a resampling process was applied, which will be further explained in Section 2.3.

Given that the study involves human participants, the relevant ethical approvals were obtained from both the Curtin University of Technology (Human Research Ethics Office): HR 12/2016 and St John of God Hospital (Human Research Ethics Committee): 823. 

### 2.3. Data Preprocessing

As stated, the gait data were collected from different participant groups over different periods of time. Therefore, the number of samples captured from the pre, post 1, post 2, and normal groups was 203, 199, 136, and 178 respectively. To resolve this data size problem, some of the long walking trials were subdivided into two walking sample sets, each set including at least three walking steps. For example, to match the number of samples in the post 2 data set, 67 long walking trials were selected and each was split into two trials. This increased the original number of post 2 samples by 67. After applying the mentioned resampling method to each data set, the number of walking samples for all groups was normalized to 203 trials.

The captured dataset consisted of three angle measurement in the format of a time series signal for pitch, roll, and yaw movement. Fast Fourier transform (FFT) was implemented on these time series signals to extract the signals’ features, such as the fundamental harmonic, amplitude, and phase shifts. Previous studies have confirmed the capability of FFT in analyzing gait and IMU data [27,35]. The signals were modelled using FFT as follows:

(1) $$\begin{array}{c} F\left(t\right)=\underset{i=0}{\sum }P_{i}\sin\left(2\pi f_{0}i+\phi_{i}\right), \end{array}$$

where $f_{0}$, $P_{i}$, and $\phi_{i}$ are the fundamental harmonic, amplitude, and phase shift of the $i^{\mathrm{th}}$ harmonic, respectively. The FFT was applied to each angle from the walking samples, meaning a 15-feature model in the frequency domain was obtained [36]. The foot sensor (S1), shank sensor (S2), and thigh sensor (S3) recorded the movements, with each sensor representing the movement in the sagittal, coronal, and transverse planes as the pitch, roll, and yaw, respectively. In addition to the 15-feature model in the frequency domain, also, each angle in the time domain constituted an extra nine features. The final model consisted of 144 features as below:

(2) $$144\;Features\left(\overset{Frequency\;domain}{\overbrace{\underset{F_{1-5}}{\underset{︸}{5}}+\underset{P_{1-5}}{\underset{︸}{5}}+\underset{\phi_{1-5}}{\underset{︸}{5}}}}+\overset{Time\;domain}{\overbrace{\underset{Angle}{\underset{︸}{1}}}}\right)\times \overset{Movments}{\overbrace{\underset{Pitch,\;Roll,\;Yaw}{\underset{︸}{3}}}}\times \overset{Sensors}{\overbrace{\underset{Thigh,\;Shank,\;Foot}{\underset{︸}{3}}}}.$$

### 2.4. Feature Extraction, Classification, and Regression

The Waikato Environment for Knowledge Analysis (WEKA) software version 3.8 was used as the workbench for evaluation of the 11 ML algorithms used to classify gait pattern based on the model with 144 features [37]. The following are the 11 classification algorithms that were analyzed: Deep learning, multilayer perceptron, K-nearest neighbors (IBK), logistic regression, Bayes net, naive Bayes, C4.5 decision tree (J48), random forest (unlimited depth with 100 iterations), random tree (unlimited depth with 100 iterations), support vector machine (SVM) (radial basis function kernel), and OneR (1R). A 10-fold cross-validation methodology was applied for each classification.

Four measures were used to compare the performance of the 11 classification algorithms. First, classification accuracy was defined as the number of correct predictions over the total number of instances in that dataset. Second, the confusion matrix, which provides information about correct and incorrect predictions, was created for each classifier [38]. The confusion matrix is a square matrix in which $C_{i,j}$ indicates the number of instances predicted as class i, where they were from class j originally. The best classification will have only zero values outside the main diagonal. In addition, the precision and F-score were calculated:

(3) $$Precision=\frac{TP}{\left(TP+FP\right)},$$

(4) $$F\;score=\frac{2\times TP}{2\times TP+FP+FN},$$

where *TP*, *FP*, and *FN* are the true positive, false positive, and false negative, respectively. True positives are items correctly labeled as belonging to their class. False positives are items incorrectly labeled as belonging to the class. False negatives are items which were not labeled as belonging to the class, but should have been. Among all 144 features describing gait in this model, some may have a higher effect in describing the severity level of foot drop. To find features with the most significant effect, the wrapper feature selection technique was implemented. The wrapper technique-based method was implemented alongside the classification algorithm to review the subset of the input features that maximizes a predefined objective function. In this case, the objective was to maximize the classification accuracy and to minimize the false alarm rate. A vector of scores for all features indicates the significance of the features. In this study, the wrapper feature selection technique was conducted using all 11 classification algorithms and the results are presented in the next section [39]. In this procedure, the data set was shuffled randomly and split into 10 groups. Each group was taken as the hold out set (or test data set) once and the remaining groups as the training data set.

Classification was done on the training set and evaluated on the test set retaining an evaluation score. The 10 fold cross-validation procedure was applied 11 times using each classification algorithm and the whole dataset was evaluated each time.

In addition to the aim of classifying gait, a further aim of this study was to find an objective index to indicate the severity of observed foot drop symptoms. To achieve this index, eight regression ML algorithms were investigated using the WEKA framework. The following are the eight regression ML algorithms that were analyzed: Deep learning, multilayer perceptron, IBK, random forest, random tree, linear regression, simple linear regression, and SVM regression. Some of the classification algorithms provide an index indicating the likelihood of their prediction, therefore they can be used as the regression algorithem. To be able to perform the regression analysis on the dataset, the state variable, which indicates in which state the sample was captured (e.g., pre, post 1, post 2), was changed to a numerical value from zero to four, which refers to the pre, post 1, post 2, and normal states, respectively.

Different measures were used to evaluate the performance of the regression algorithms. The first measure was the error between the predictions and the actual value of the class. For example, a regression algorithm may have predicted a post 2 (i.e., 2) walking sample as 2.8, and therefore the error for this sample is 0.8. Additionally, the correlation coefficient, mean absolute error, root mean square error (RMSE), relative absolute error, and root relative squared error were used as measures for evaluating the regression performance as shown below:

(5) $$Correlation\;coefficient=\frac{N\sum y\overset{´}{y}-(\sum y)(\sum \overset{´}{y})}{\sqrt{\left[N\sum y^{2}-{\left(\sum y\right)}^{2}\right]\left[N\sum {\overset{´}{y}}^{2}-{\left(\sum \overset{´}{y}\right)}^{2}\right]}},$$

(6) $$Mean\;absolute\;error=\frac{1}{N}\overset{N}{\underset{i=1}{\sum }}\left|y-\overset{´}{y}\right|,$$

(7) $$Root\;mean\;absolute\;error=\sqrt{\frac{1}{N}\overset{N}{\underset{i=1}{\sum }}{\left(y-\overset{´}{y}\right)}^{2}},$$

(8) $$Relative\;absolute\;error=\frac{\sum_{i=1}^{N}\left|y_{i}-\overset{´}{y_{i}}\right|}{\sum_{i=1}^{N}\left|\bar{y}-\overset{´}{y_{i}}\right|}\times 100,$$

(9) $$Root\;relative\;squared\;error=\sqrt{\frac{\sum_{i=1}^{N}{\left(\overset{´}{y_{i}}-y_{i}\right)}^{2}}{\sum_{i=1}^{N}{\left(\overset{´}{y_{i}}-\bar{y}\right)}^{2}}}\times 100,$$

where y and $\overset{´}{y}$ are the actual and prediction values and N is the number of samples.

## 3. Results

This section compares the performance of the ML algorithms on the collected data. 

### 3.1. Analysis of the Four Classes

First, the gait patterns were classified into four classes using the entire available dataset from the patients at different stages of treatment and the participants in the normal group. These four classes were the pre, post 1, post 2, and normal class.

Table 1 presents the accuracy of the ML classification algorithms for the four classes. 

**Figure 1:**
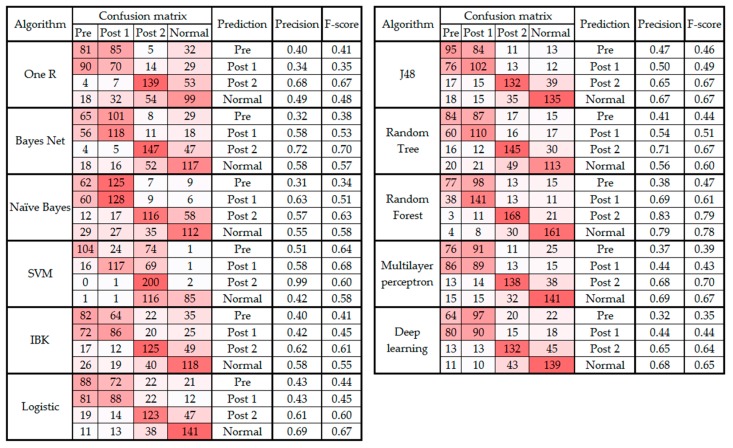
Confusion matrix, precision, and F-score from classifying data into four classes.

Table 1 demonstrates that random forest and OneR have the maximum and minimum accuracy, respectively. The average overall accuracy of the algorithms is 55.34%, which is low. To investigate the cause of the low accuracy of the algorithms, the confusion matrix was generated and investigated. Figure 1 presents the confusion matrix, precision, and F-score observed from all classification algorithms. The figure is color coded so that as the value of the cell increases, the cell is colored with a darker red.

In addition to the classification algorithms, the regression method was used to compare and evaluate each class data on a numerical basis. Here, the pre category was given a base value of 1, post 1 a value of 2, post 2 a value of 3, and normal a value of 4. Table 2 presents the error measures of the eight regression algorithms for the four classes (i.e., pre, post 1, post 2, and normal).

**Figure 2:**
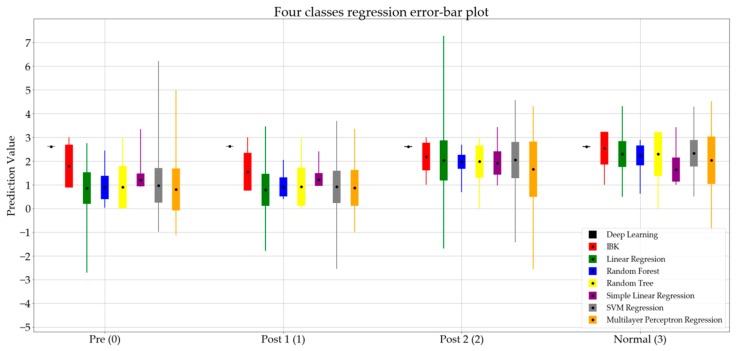
Error bar plot for regression algorithms classifying data into four classes.

Figure 2 presents the error of the predictions and the error bar plot of the regression algorithms.

In Figure 2, the mean value of the prediction of each algorithm is represented by a black dot, while the standard deviation is represented by a rectangle. The minimum and maximum values are presented using thin lines in each bar. Figure 2 represents the eight regression algorithms for the four classes.

It is notable in Figure 1 that in all 11 classifications, the predictions for the pre and post 1 categories are often confused (i.e., the data from the pre class were classified as post 1 and vice versa). This error in prediction is the principal cause of the low accuracy level of the algorithms presented in Table 1. Therefore, the following section presents the results from the classification and regression algorithms on the dataset without post 1 data.

### 3.2. Analysis of Three Classes

This section presents the results of the classification tests when the post 1 class was removed from the analysis. Table 3 presents the accuracy of ML algorithms when the dataset was classified into three classes of pre, post 2, and normal. The second column shows the classification results when all 144 features were used. In general, Table 3 compares the accuracy observed before and after applying the wrapper feature selection technique.

**Figure 3:**
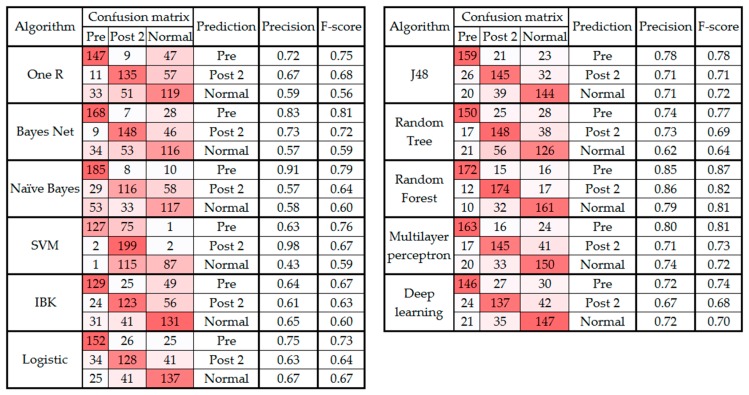
Confusion matrix, precision, and F-score from classification algorithms classifying data into three classes before feature selection.

**Figure 4:**
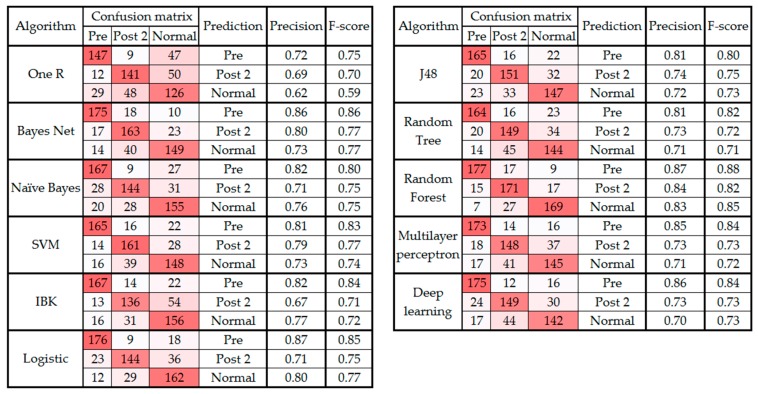
Confusion matrix, precision, and F-score from classification algorithms classifying data into three classes after feature selection.

Figure 3 and Figure 4 present the confusion matrix, precision, and F-score of the classification algorithms before and after the feature selection, respectively.

As seen in Table 3, the classification performance improved after applying feature selection. The best performing classifier was random forest before and after feature selection. Also, the random forest as the best classifier had 33 selected features (Table 3) using the wrapper technique. Table 4 indicates the type and body part of the selected features.

For the next step of the analysis, the regression algorithms were applied to the three classes (i.e., pre, post 2, and normal), and the regression error measures calculated are presented in Table 5.

**Figure 5:**
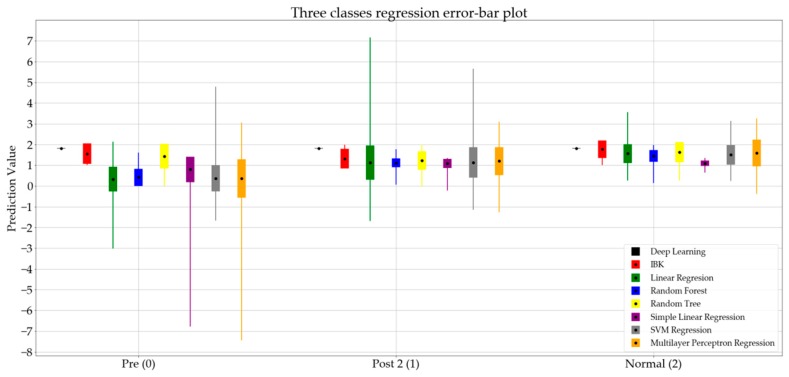
Error bar plot for eight regression algorithms classifying data into three classes.

Figure 5 presents the error bar plot of the regression algorithms. In this figure, the pre, post 2, and normal states are represented by the numbers 0, 1, and 2, respectively.

## 4. Discussion

This research demonstrated a systematic and objective methodology for the evaluation of foot drop with L5 lumber radiculopathy origins. 

As presented in Table 5, the random forest regression shows the lowest mean absolute error. After investigating the performance of the random forest regression more closely in Figure 5, and comparing the results for the pre and post 2 states, a jump in the mean value of the predictions is noticeable. This indicates that the prediction values for the random forest algorithm can be used as an index to determine the severity level of foot drop in the walking gait pattern. Therefore, the method presented in this research shows promise as a potential measurement tool for tracking the recovery process of foot drop with L5 origins in adults. However, outliers in the random forest algorithm require further investigation.

This study found that the random forest algorithm provides the best classification, with an 84.89% accuracy. The average accuracy of the classifiers improved significantly after removing the post 1 class and applying the classification to three instead of four classes. Additionally, the use of wrapper feature selection proved to be effective in improving the classification performance of the algorithms in the three class analysis (Table 3). The improvements in accuracy when decreasing the number of features indicates that the current IMU system can be simplified by reducing the number of sensors, which will lower the computation expenses. Also, Table 4 indicates that the wrapper technique, which was applied to the random forest classifier, selected features mainly from the foot and shank regions, demonstrating the correlation between the IMU sensor location and the ability to classify foot drop conditions. In addition, 48.5% of the all selected features were from pitch (flexion) movement that is known to be affected by foot drop.

As noted in Section 3 and presented in Figure 1, the confusion matrix of all the classification algorithms revealed similarity in the gait patterns of the pre and post 1 groups, which led to confusion between these two classes of movement. This raises an important question about the timeline of monitoring foot drop patients after lumbar spine surgery. According to Section 3, the improvement can be fully observed at least two to three weeks after the surgery [40]. In addition, the confusion matrixes before and after feature selection (Figure 3 and Figure 4) showed that the highest level of confusion occurs between the post 2 and normal stages, which indicates that two to three weeks after surgery, the walking patterns of the patients are similar to the walking patterns of people who are not suffering from foot drop (i.e., the normal group). Also, referring to the F1 scores in Figure 1 and Figure 3, it is noticeable that the false positive or false negative rates are highly reduced while remaining cases can be addressed in the clinical environment by using simultaneous assessments.

In this study, it was demonstrated that the ML algorithms are capable of classifying patients with foot drop from normal patients, without any knowledge of specific gait events (i.e., swing phase, heel contact, toe-off, etc.). This is beneficial since no extra steps are required to identify gait events before the application of ML algorithms.

Figure 2 summarizes the prediction values of the regression algorithms. Comparing the standard deviation for the normal set among the four groups, it is notable that most of the algorithms show a smaller standard deviation for the normal group, which indicates the diversity of gait patterns at different stages of spinal surgery treatment. 

In addition to the work in this study, the proposed system has the potential to be used in the clinical environment for an objective evaluation and assessment of gait in the case of any gait-related disorder. While this study only investigated foot drop patients with L5 origins, the study’s approach can be applied to any other group of patients with gait-related disorders, such as children with cerebral palsy [41,42]. 

The system presented in this study has the potential to be used for long-term patient monitoring at home, which not only helps to enable continuous tracking of patient recovery, but also provides more gait data that can help to improve the accuracy of the analysis.

In addition, the system could be used to track the walking pattern of both legs of one patient to compare the gait patterns of both the left and right leg of a patient. 

## 5. Conclusions

This study tested the feasibility of an IMU system with ML analysis to assess the level of severity in foot drop patients by utilizing an applicable method in clinical environments. An application of this approach is to evaluate gait conditions and track the recovery of gait disorders, such as foot drop. The study applied two ML approaches to achieve its aim: Classification and regression. In each approach, multiple ML algorithms were evaluated and compared over the datasets of walking gait from a sample of healthy participants with normal gait styles and a group of patients with foot drop in different stages of lumbar spine surgery. The evaluation was based on the accuracy, confusion matrix, and mean absolute error of the algorithms after classification of the different characteristics of the gaits of participants. The random forest classifier initially resulted in the best accuracy (83.25%). The application of the wrapper feature selection technique to the random forest algorithm improved the accuracy to 84.89%.

## Figures and Tables

**Table 1:** Accuracy from 11 ML algorithms classifying data into four classes.

Algorithm	Accuracy (%)
Bayes net	55.0493
Deep learning	52.3399
IBK	50.6158
J48	57.1429
Logistic regression	54.1872
Multilayer perceptron	54.6798
Naïve Bayes	51.4778
OneR	47.9064
Random forest	67.3645
Random tree	55.665
SVM	62.3153
Average	55.3400

**Table 2:** Error measures from eight regression algorithms classifying data into four classes.

Algorithm	Correlation Coefficient	Mean Absolute Error	Root Mean Squared Error	Relative Absolute Error	Root Relative Squared Error
Deep learning	–0.0856	1.3104	1.5825	130.8814	141.4032
IBk	0.4181	0.7808	1.2217	77.9844	109.1655
Linear regression	0.6430	0.7003	0.8910	69.9453	79.6130
Multilayer perceptron	0.0026	1.2089	9.3257	120.7437	833.2870
Random forest	0.7807	0.5426	0.7119	54.1923	63.6136
Random tree	0.5748	0.6252	1.0010	62.4422	89.4466
Simple linear regression	0.4454	0.7936	1.0010	79.2604	89.4430
SVM	0.6244	0.7062	0.9067	70.5317	81.0133

**Table 3:** Classification accuracy from 11 ML algorithms classifying data into three classes before and after feature selection.

Algorithm	All Features (n = 144)	Selected Features	
	Accuracy (%)	Accuracy (%)	Number of Selected Features
Bayes net	70.94	79.97	20
Deep learning	70.61	76.52	21
IBk	62.89	75.37	46
J48	73.56	76.03	22
Logistic regression	68.47	79.15	22
Multilayer perceptron	75.21	76.52	20
Naïve Bayes	68.64	76.52	44
OneR	65.85	67.98	1
Random forest	83.25	84.89	33
Random tree	69.62	75.04	21
SVM	67.82	77.83	30
Average	70.62	76.89	NA

**Table 4:** The features selected by wrapper techniques using the random forest algorithm.

Feature Type	Foot	Shank	Thigh	Total
Pitch	5	5	6	16
Roll	6	4	1	11
Yaw	4	1	1	6
**Total**	15	10	8	33

**Table 5:** Error measures from eight regression algorithms classifying data into four classes.

Algorithm	Correlation Coefficient	Mean Absolute Error	Root Mean Squared Error	Relative Absolute Error (%)	Root Relative Squared Error (%)
Deep learning	−0.0598	0.9396	1.1555	140.4993	141.4422
IBk	0.4966	0.4631	0.8245	69.2432	100.9249
Linear regression	0.6109	0.5319	0.5319	79.5453	88.4125
Random forest	0.7931	0.3785	0.5099	56.6055	62.4162
Random tree	0.5861	0.3992	0.7267	59.6992	88.9477
Simple linear regression	0.2998	0.6580	0.7963	98.3908	97.4729
SVM	0.5930	0.5470	0.7221	81.8015	88.3848
Multilayer perceptron	0.5469	0.5649	0.8279	84.4696	101.3445
